# Sox9 Determines Translational Capacity During Early Chondrogenic Differentiation of ATDC5 Cells by Regulating Expression of Ribosome Biogenesis Factors and Ribosomal Proteins

**DOI:** 10.3389/fcell.2021.686096

**Published:** 2021-06-21

**Authors:** Marjolein M. J. Caron, Maxime Eveque, Berta Cillero-Pastor, Ron M. A. Heeren, Bas Housmans, Kasper Derks, Andy Cremers, Mandy J. Peffers, Lodewijk W. van Rhijn, Guus van den Akker, Tim J. M. Welting

**Affiliations:** ^1^Laboratory for Experimental Orthopedics, Department of Orthopedic Surgery, CAPHRI Care and Public Health Research Institute, Maastricht University Medical Center, Maastricht, Netherlands; ^2^Maastricht MultiModal Molecular Imaging Institute (M4I), Division of Imaging Mass Spectrometry, Maastricht University Medical Center, Maastricht, Netherlands; ^3^Department of Clinical Genetics, Maastricht University Medical Center, Maastricht, Netherlands; ^4^Department of Musculoskeletal Biology, Institute of Life Course and Medical Sciences, University of Liverpool, Liverpool, United Kingdom

**Keywords:** ATDC5, chondrogenesis, Sox9, ribosome, translation, proteomics, transcriptomics

## Abstract

**Introduction:**

In addition to the well-known cartilage extracellular matrix-related expression of Sox9, we demonstrated that chondrogenic differentiation of progenitor cells is driven by a sharply defined bi-phasic expression of Sox9: an immediate early and a late (extracellular matrix associated) phase expression. In this study, we aimed to determine what biological processes are driven by Sox9 during this early phase of chondrogenic differentiation.

**Materials:**

Sox9 expression in ATDC5 cells was knocked down by siRNA transfection at the day before chondrogenic differentiation or at day 6 of differentiation. Samples were harvested at 2 h and 7 days of differentiation. The transcriptomes (RNA-seq approach) and proteomes (Label-free proteomics approach) were compared using pathway and network analyses. Total protein translational capacity was evaluated with the SuNSET assay, active ribosomes were evaluated with polysome profiling, and ribosome modus was evaluated with bicistronic reporter assays.

**Results:**

Early Sox9 knockdown severely inhibited chondrogenic differentiation weeks later. Sox9 expression during the immediate early phase of ATDC5 chondrogenic differentiation regulated the expression of ribosome biogenesis factors and ribosomal protein subunits. This was accompanied by decreased translational capacity following Sox9 knockdown, and this correlated to lower amounts of active mono- and polysomes. Moreover, cap- versus IRES-mediated translation was altered by Sox9 knockdown. Sox9 overexpression was able to induce reciprocal effects to the Sox9 knockdown.

**Conclusion:**

Here, we identified an essential new function for Sox9 during early chondrogenic differentiation. A role for Sox9 in regulation of ribosome amount, activity, and/or composition may be crucial in preparation for the demanding proliferative phase and subsequent cartilage extracellular matrix production of chondroprogenitors in the growth plate *in vivo*.

## Introduction

Chondrogenesis, or chondrogenic differentiation, is the differentiation path of progenitor cells *via* early mesenchymal condensation into chondrocytes that synthesize a cartilaginous extracellular matrix (ECM) (Kronenberg, 2003; Lefebvre and Smits, 2005; Mackie et al., 2008). Aside from formation of articular cartilage and its maintenance, skeletal development also depends on chondrogenic differentiation. Development of the long bones of the mammalian skeleton depends on the activity of growth plates, cartilaginous entities at the ends of developing bones in which chondrocytes differentiate from progenitor cells (Kronenberg, 2003; Lefebvre and Smits, 2005; Mackie et al., 2008). In contrast to articular chondrocytes, differentiating growth plate chondrocytes are predestined to undergo hypertrophic differentiation and apoptosis. The remaining cartilaginous matrix is subsequently remodeled by osteoclastic/osteocytic activity, resulting in *de novo* synthesized bone tissue (Kronenberg, 2003; Lefebvre and Smits, 2005; Mackie et al., 2008). *In vivo*, chondrogenic differentiation is almost exclusively initiated from local mesenchymal progenitor cells that reside in the cartilaginous tissue [growth plate resting zone (Abad et al., 2002), articular cartilage superficial layer (Karlsson and Lindahl, 2009)] or in surrounding fibrous tissues [e.g., periosteum (Nakahara et al., 1990; Emans et al., 2011)]. However, *in vitro* chondrogenic differentiation has been reported from various primary (mesenchymal) progenitor cell sources including synovial membrane/fluid, bone marrow, adipose tissue, fibroblasts, and induced pluripotent stem cells (Barry and Murphy, 2004; French et al., 2004; Medvedev et al., 2011). In addition to high amounts of oligosaccharides (mostly hyaluronic acid, heparan sulfate, and chondroitin sulfate), important cartilage ECM proteins are type II collagen (Col2a1) and aggrecan (Acan) (Mankin et al., 2000; de Crombrugghe et al., 2001; Lefebvre and Smits, 2005).

The master regulator of chondrogenic differentiation is the transcription factor SRY (sex determining region Y)-box 9 (Sox9). Mutations in *SOX9* were originally identified as the cause for campomelic dysplasia (Foster et al., 1994; Wagner et al., 1994), a severe skeletal dysplasia associated with XY sex reversal and disproportionally short stature, as well as general lack of cartilaginous tissue formation. *SOX9* was found to be essential for murine early chondrogenic lineage determination (Akiyama et al., 2002). Upon nuclear translocation (Argentaro et al., 2003; Haudenschild et al., 2010), Sox9 binds as a homodimer to its consensus DNA recognition sequence (A/T) (A/T)CAA(A/T)G (Mertin et al., 1999), which includes the highly conserved AACAAT motif recognized by the HMG-box domain shared among Sox and Sry protein family members. In chondrogenic differentiation, Sox9 drives the transcription of, and cooperates with, L-Sox5 and Sox6 for efficient transcription of the *COL2A1* and *ACAN* genes (Lefebvre et al., 1997, 1998, 2001; Akiyama et al., 2002; Han and Lefebvre, 2008). Other cartilage ECM genes have also been demonstrated as under transcriptional control of SOX9, including *COL9A1* (Genzer and Bridgewater, 2007), *COL27A1* (Jenkins et al., 2005), and *MATN1* (Rentsendorj et al., 2005; Oh et al., 2010). Besides L-Sox5 and Sox6, another important factor for Sox9-mediated transcription is Smad3. Smad3 modulates the interaction between Sox9 and CBP (CREB-binding protein)/p300 (Furumatsu et al., 2005), thereby possibly explaining the pro-chondrogenic effect of bone morphogenetic proteins (BMPs) and transforming growth factor beta (TGFβs) on chondrogenic differentiation (Yoon and Lyons, 2004; Wang et al., 2014).

During chondrogenic differentiation of progenitor cells *in vitro*, induction of Sox9 expression is biphasic (Caron et al., 2012). In the first hours after initiation of chondrogenic differentiation, Sox9 expression is transiently induced (immediate early Sox9 induction), together with the other members of the “Sox-trio.” Sox9 expression increases a second time, in parallel with the synthesis of cartilage ECM molecules (late Sox9 induction). Previously, we demonstrated that this immediate early Sox9 expression is in part regulated by the immediate early response gene 1 (Egr1) (Spaapen et al., 2013) as well as by NFκB/p65 (Caron et al., 2012). Similar expression patterns were also found in growth plate sections (Caron et al., 2012). The function of the early Sox9 induction itself remains elusive. In the present work, we therefore determined the transcriptomic and proteomic consequences of the abrogation of early Sox9 expression during ATDC5 chondrogenic differentiation, and uncovered the biological processes that are driven by Sox9 during the early phase of chondrogenic differentiation.

## Materials and Methods

### ATDC5 Cell Culture

ATDC5 cells (RIKEN BRC, Japan, STR profiled) (Atsumi et al., 1990) were cultured in a humidified atmosphere at 37°C and 5% CO_2_ in culture media consisting of Dulbecco’s Modified Eagle Medium (DMEM)/F12 (Life Technologies, Waltham, MA, United States), 5% fetal calf serum (Life Technologies), 1% antibiotic/antimycotic (Life Technologies), and 1% non-essential amino acids (NEAA) (Life Technologies). Chondrogenic differentiation was induced by plating the cells in triplicates at 6,400 cells/cm^2^, or 20,000 cells/cm^2^ in transfection experiments, and the addition to culture media of differentiation supplements 10 μg/ml insulin (Sigma-Aldrich, St. Louis, MO, United States), 10 μg/ml transferrin (Roche, Basel, Switzerland), and 30 nM sodium selenite (Sigma–Aldrich). Media was refreshed every 2 days.

### Sox9 Loss and Gain of Function

A small interfering RNA (siRNA) duplex for Sox9 (“Sox9 RNAi”) (sense: 5′-GACUCACAUCUCUCCUAAUTT-3′, anti-sense: 5′-AUUAGGAGAGAUGUGAGUCTT-3′) and a scrambled siRNA duplex (“Control RNAi,” Eurogentec, Seraing, Belgium) were transfected (100 nM) 1 day prior to initiation of chondrogenic differentiation or at day 6 of differentiation using HiPerFECT according to manufacturers’ protocol (Qiagen, Hilden, Germany). A custom-made DNA strand containing a start codon and 3×FLAG sequence (derived from p3×FLAG CMV7.1) and the mSox9 coding sequence (NM_011448.4:376-1899) without start codon was flanked by 5′*Eco* RI and 3′*Xba* I restriction sites (GeneCust, Boynes, France). This fragment was cloned directionally into the pLVX-EIF1α-IRES puro MCS (Takara, Saint-Germain-en-Laye, France) to generate a pLVX-EIF1α-mSox9-IRES puro transfer plasmid. Lentiviral particles were generated according to manufacturer’s instructions with the fourth-generation VSV-G envelope Lenti-X system (Takara, Saint-Germain-en-Laye, France). Lentiviral titers were determined by p24 ELISA (enzyme-linked immunosorbent assay; INNOTEST HIV antigen mAb, Fujirebio, Zwijnaarde, Belgium). Viral transductions were performed by incubation of 1 ng lentivirus/cell in the presence of 8 μg/ml polybrene (Sigma-Aldrich) for 8 h, followed by an overnight incubation with 1.6 μg/ml polybrene.

### RNA Isolation

RNA was isolated using TRIzol (Life Technologies), collecting the aqueous phase after centrifugation. RNA was precipitated with isopropanol (VWR International, Radnor, PA, United States) (−80°C) and pellet by centrifugation. RNA pellets were washed in 80% ethanol (VWR International) and dried. RNA was dissolved in DNase/RNase-free pure water. RNA quantity and purity were determined spectrophotometrically (Biodrop, Isogen Life Sciences, Utrecht, Netherlands).

### Quantitative Real-Time PCR

Total RNA was reverse transcribed into cDNA using standard procedures and random hexamer priming as previously described (Welting et al., 2011). Real-time quantitative PCR (RT-qPCR) was performed using Mesagreen qPCR master mix plus for SYBR Green (Eurogentec, Liège, Belgium). A CFX96 Real-Time PCR Detection System (Bio-Rad, Hercules, CA, United States) was used for amplification: initial denaturation 95°C for 10 min, followed by 40 cycles of amplification (denaturing 15 s at 95°C and annealing 1 min at 60°C). Validated primer sequences are shown in Supplementary Table 1. Data were analyzed using the standard curve method, mRNA expression was normalized to a reference gene, and gene expression was calculated as fold change as compared to control conditions or *t* = 0.

### RNA Sequencing and Analysis

Isolated RNA was checked for quality and integrity on the Agilent 2100 Bioanalyzer (Santa Clara, CA, United States) *via* 2100, an Expert Eukaryote Total RNA Nano chip according to the manufacturer’s protocol. The mRNA sequencing library was generated using TruSeq mRNA sample preparation kit (Illumina, Eindhoven, Netherlands). In short, mRNA was enriched using magnetic beads coated with poly-dT, followed by fragmentation. The fragmented mRNA-enriched samples were subjected to cDNA synthesis by reverse transcriptase, followed by dA-tailing and ligation of specific double-stranded bar-coded adapters. Next, the library was amplified, and following cleanup, the sizes of the libraries were determined on an Agilent 2100 Bioanalyzer *via* a DNA 1000 chip according to the manufacturer’s protocol. Pooled libraries consisting of equal molar samples were sequenced on a high-output 75-bp single read on the NextSeq500 (Illumina). For each sample, the number of reads covering one or more exons of a given transcript were extracted. Triplicates of samples that were treated with either Scrambled or Sox9 siRNAs, at two different time points, were grouped separately. A transcript was defined as expressed when all replicates of a group had at least five reads extracted within the transcript’s region. The grouped data were then compared to one another. The fold-change difference and the *p*-value were calculated using R-package edgeR (Robinson et al., 2010; McCarthy et al., 2012), after which the *p*-value was corrected for multiple testing [false discovery rate (FDR)-corrected]. Transcripts having an FDR-corrected *p*-value < 0.05 and a fold change of at least 1.5 were considered differentially expressed transcripts. RNA-seq data have been deposited in the ArrayExpress database at EMBL-EBI^1^ under accession number E-MTAB-10333. EnrichR (Chen et al., 2013) software was used to display the pathways of interest obtained from the enrichment of down- or upregulated proteins. The top three pathways of interest were considered from both Wikipathways and KEGG software (version 2019, Mouse) based on the combined score of the *p*-value and the adjusted *p*-value scores.

### Label-Free Proteomics

At indicated time points, plates were rinsed three times with 1% phosphate buffered saline (PBS). A mixture containing complete Mini Protease Inhibitor Cocktail (Roche, Basel, Switzerland) in 25 mM ammonium bicarbonate (ABC) buffer (Sigma-Aldrich, Zwijndrecht, Netherlands) and 6M urea (GE Healthcare, Eindhoven, Netherlands) was added to the plates. Cells were collected by scraping with a rubber policeman and the samples were transferred to Eppendorf tubes. Triplicates were pooled and sonicated for 10 min and centrifuged at 12,000*g* for 10 min in 4°C. The supernatant containing proteins was transferred into new tubes and a Bradford assay (Bio-Rad, Lunteren, Netherlands) was performed to assess protein concentration. The concentrations were adjusted to 0.2 μg/μl in order to normalize for the following steps. Samples were reduced with 20 mM of dithiothreitol (DTT) (Sigma-Aldrich) for 45 min and alkylated with 40 mM of iodoacetamide (IAM; Sigma-Aldrich) for 45 min in the dark. The alkylation step was stopped by adding 20 mM of DTT. Samples were then digested using LysC and trypsin (Promega, Leiden, Netherlands) added at a ratio of 1:25 (enzyme:protein) and incubated for 2 h at 37°C in a water bath. Finally, 200 μl of 25 mM ABC buffer was added to the samples before overnight incubation at 37°C. The digestion was stopped by adding formic acid (FA; Sigma-Aldrich) and acetonitrile (Biosolve) at a final concentration of 1 and 2%, respectively. Two hundred nanograms of each sample were injected in duplicate for liquid chromatography mass spectrometry (LC-MS/MS) analysis. The separation of the peptides was performed on a Thermo Fisher Scientific Dionex Ultimate 3000 Rapid Separation ultrahigh-performance liquid-chromatography (HPLC) system (Thermo Scientific, MA, United States) equipped with an Acclaim PepMap C18 analytical column (2 μm, 75 μm^∗^150 mm, 100 Å). The samples were first trapped on an online C18 column for desalting. The peptides were then separated on the analytical column with a 90-min linear gradient from 5 to 35% acetonitrile/0.1% FA and a flow rate set at 300 nl/min. The HPLC system was coupled to a high mass resolution Orbitrap MS instrument (Q-Exactive HF, Thermo Scientific, Waltham, MA, United States). The mass spectrometer was operated in data-dependent acquisition (DDA) mode with the following settings: Full MS scan of the mass range *m/z* 350–1,650 at a resolution of 120,000 at *m/z* 400, followed by tandem mass spectrometry (MS/MS) scans for the fragmentation of the 15 most intense ions at a resolution of 30,000. The ions already selected for fragmentation were dynamically excluded for 20 s. External calibration of the instrument was performed using a standard calibration solution for positive ion mode (Thermo Scientific). For protein identification, raw files were processed within the Proteome Discoverer software version 2.2 (Thermo Scientific) using the search engine Sequest with the Swiss-Prot database *Mus musculus* version 2017-10-25 (TaxID 10090). The following parameters were used for the database search: carbamidomethylation of Cysteine for fixed modifications; oxidation of Methionine and acetylation of protein N-terminal for variable modifications; trypsin for enzyme with a maximum of two missed cleavages; y and b for the ion types with a mass tolerance of 10 ppm and 0.02 Da for the precursors and the fragments, respectively; minimum and maximum peptide length of 6 and 144, respectively. Normalization of the data was performed on the total peptide amount. Percolator was used for the decoy database search and the FDR was fixed at 1% maximum. Finally, a list of 23 commonly detected contaminants were removed manually (data not shown). For protein quantitation, the Minora Feature Detector node in the processing step and the Feature Mapper node combined with the Precursor Ions Quantifier node in the consensus step were used with default settings. The mass spectrometry proteomics data have been deposited to the ProteomeXchange Consortium *via* the PRIDE (Perez-Riverol et al., 2019) partner repository with the dataset identifier PXD024715. ANOVA test and principal component analysis (PCA) were performed within the Proteome Discoverer software. PCA was performed to visualize protein abundance changes between groups in an unsupervised manner. ANOVA test was used to analyze the statistical significance of variation observed in protein abundances between the conditions. The proteins were considered modulated with a *p*-value ≤ 0.05 and a fold change (FC) ≥ 2. The modulated proteins were then imported within the EnrichR software (Chen et al., 2013) to display the top 3 pathways of down- or upregulated proteins ranked by the combined score. WikiPathways and KEGG were used as databases (version 2019, Mouse).

### Immunoblotting

**FIGURE 1:**
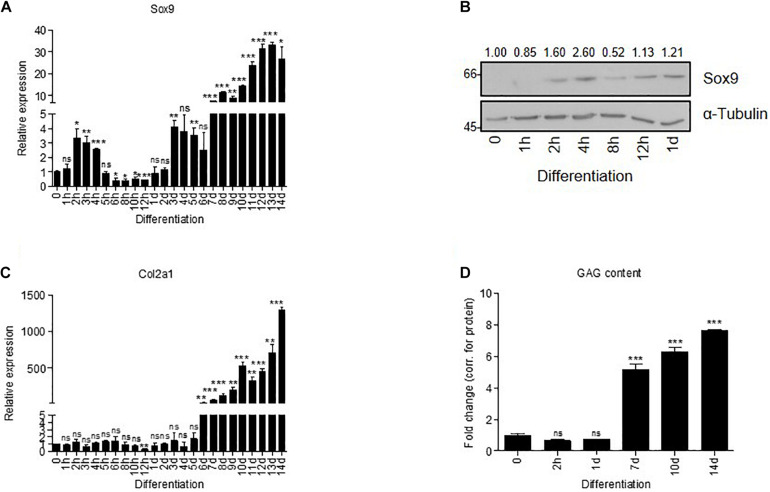
Sox9 expression has a bi-phasic peak expression during chondrogenic differentiation of ATDC5 cells. **(A)** Sox9 mRNA expression showed bi-phasic peak pattern during ATDC5 differentiation (h = hours, d = days) as measured by RT-qPCR (Caron et al., 2012; Spaapen et al., 2013). Results were normalized to β-Actin mRNA expression and presented relative to *t* = 0. **(B)** Sox9 protein expression peak during early ATDC5 differentiation as measured by immunoblotting. α–Tubulin was used as loading control. Molecular weight markers (in kDa) are shown on the left and relative quantifications are depicted on top of immunoblot. **(C)** In similar samples from **(A)**, Col2a1 mRNA expression was measured during ATDC5 differentiation by RT-qPCR and showed an increase in expression from day 6 onward. **(D)** GAG content (by Alcian Blue staining and corrected for total protein expression) was determined during ATDC5 differentiation. Experiments were performed in triplicate, bars represent mean ± SEM. ns = not significant, *p < 0.05, **p < 0.01, ***p < 0.0001.

Cells were lysed in RIPA buffer [150 mM NaCl, 1% NP-40, 0.5% sodium deoxycholate, 0.1% SDS, 50 mM Tris, pH 8.0, 5.0 mM ethylenediaminetetraacetic acid (EDTA), pH 8.0, 0.5 mM dithiothreitol (DTT) supplemented with cOmplete Mini Protease Inhibitor Cocktail (Roche) and PhosSTOP phosphatase inhibitor (Sigma-Aldrich)]. Extracts were sonicated and protein concentrations were determined with a bicinchoninic acid (BCA) assay (Sigma-Aldrich). Proteins were separated by SDS-PAGE [sodium dodecyl (lauryl) sulfate-polyacrylamide gel electrophoresis] and transferred to nitrocellulose membranes by electroblotting. Primary antibodies for immunodetection were anti-Sox9 (Abcam, Cambridge, United Kingdom; ab3697) and anti-Tubulin (Sigma-Aldrich; T6074). Bound primary antibodies were detected using immunoglobulins conjugated with HRP (horseradish peroxidase; DakoCytomation, Glostrup, Denmark) and visualized by enhanced chemoluminescence (ECL). ECL signals were quantified using ImageJ 1.46f software (Figure 1B). Relative differences in Sox9 levels, corrected for background and Tubulin levels, were determined as compared to *t* = 0 conditions.

### sGAG Assay

The sulfated glycosaminoglycan (GAG) content was measured using a modified dimethyl methylene blue (DMB) assay (Farndale et al., 1982). The absorbance of samples was read at 540 and 595 nm using a spectrophotometer (Multiskan FC, Life Technologies) and GAG concentrations were calculated using a chondroitin sulfate standard curve (Sigma-Aldrich) and corrected for total protein content using a BCA assay.

### SUnSET Assay

Protein translational capacity of ATDC5 cultures (in sextuplicates) was assessed with the SUnSET assay (Goodman and Hornberger, 2013; Henrich, 2016). Puromycin (5.4 μM; Sigma-Aldrich) was incubated for 15 min in the cell culture medium, immediately followed by washing in PBS and fixation for 20 min with 10% formalin (VWR, Radnor, PA, United States). Permeabilization was performed for 10 min with PBS supplemented with 0.1% Triton X-100. Wells were rinsed with PBS with 0.1% Tween (PBS-T) and blocked for 1.5 h with 1% (m/v) skimmed milk powder (ELK, Campina, Zaltbommel, Netherlands) in PBS-T, followed by overnight incubation at 4°C with the primary anti-puromycin antibody 12D10 (Sigma-Aldrich). After washing with PBS-T, wells were incubated for 1 h at room temperature with the secondary goat anti-mouse Alexa488 antibody (Life Technologies). The fluorescent signal intensity was determined using a TriStar^2^ LB942 (Berthold, Bad Wildbad, Germany) equipped with excitation filter F485 and emission filter F535. Fluorescent data were normalized to DNA content from the same well (McCaffrey et al., 1988). To this end, wells were washed with HEPES-Buffered Saline (HBS), followed by 1 h incubation with 5 μg/ml DAPI (Life Technologies) plus 5 μg/ml HOECHST 33342 (Life Technologies) in HBS. After subsequent washing steps with HBS, fluorescent signal intensity was determined using a TriStar^2^ LB942 (Berthold), using the excitation filter F355 and emission filter F460.

### Polysome Fractionation

Polysome fractionation was performed as described previously (Panda et al., 2017). Three 15-cm plates with ATDC5 cells were used to generate a single sample. At the day of sample collection, cells were differentiated for 2 h, then pre-treated for 5 min with 100 μg/ml Cycloheximide (Sigma), washed twice in 0.9% NaCl with Cycloheximide, and collected by scraping with a rubber policeman in cold 0.9% NaCl. Pelleted cells were lysed for 10 min in 1.8 ml of polysome extraction buffer [20 mM Tris–HCl (pH 7.5), 100 mM KCl, 5 mM MgCl_2_, 0.5% Non-idet P-40, 100 μg/ml Cycloheximide, complete protease inhibitor cocktail (Roche), and RNasin (Promega, 40 U/ml)] on ice. Nuclei and cellular debris were removed by centrifugation at 12,000 × *g* for 10 min at 4°C and 9/10th of the total volume was transferred to fresh tubes and measured spectrophotometrically. Total yield was the same for siCtrl- and siSox9-treated cells. Sucrose gradients (linear 10–50%) were made using the Gradient Master (BioComp) in ultracentrifuge tubes (Seton, SW41 tubes). Cytoplasmic extracts (250 μg/sample) were loaded to each gradient in a fixed volume (400 μl). Gradients were run on an ultra-centrifuge (Beckman L60, Brea, CA, United States) at 39,000 rpm for 1.5 h at 4°C with max acceleration and deceleration 9. Samples were fractionated into 24 × 0.5-ml fractions using a Piston Gradient fractionator (BioComp, Fredericton, Canada) and fraction collector (Gilson FC203B, Middleton, WI, United States) with continuous A260 monitoring (Triax FC-1).

### Bicistronic Reporter Assay

Reporter constructs for the CrPv IGR IRES, the CrPv CCGG IGR IRES mutant, the HCV, and the P53 IRES were a kind gift of Dr. S. Thompson (UAB, United States). One day post-plating, maxi-prep DNA (0.5 μg/well) and 100 nM siRNA were transfected into 24-well plates (*n* = 3/group) using Mirus Transit-X2 according to the manufacturer’s instructions. The next day, differentiation was induced for 24 h, and samples were collected by washing cells with 0.9% NaCl and incubation in 100 μl of passive lysis buffer for 15 min (Promega). Subsequently, samples were transferred to Eppendorf tubes and centrifuged for 10 min at 12,000 × *g* in a tabletop centrifuge. Next, 50 μl of lysate was used for dual luciferase measurements (Promega) using a Berthold injection system (Reeuwijk, Netherlands; 10 s counting time per cistron). Data are represented as fold change of the ratio Fluc/Rluc in control cells for each IRES.

### Statistics in Other Than Proteomics or Transcriptomic Analysis

Statistical significance was determined by two-tailed Student’s *t* tests using GraphPad PRISM 5.0 (La Jolla, CA, United States). Error bars in graphs represent mean ± standard error of the mean. Significance for all tests was set at *p* ≤ 0.05.

## Results

### Early Sox9 Peak in ATDC5 Chondrogenic Differentiation

Induction of Sox9 expression is biphasic during chondrogenic differentiation of progenitor cells *in vitro* (Caron et al., 2012; Spaapen et al., 2013). In the first (Abad et al., 2002; Lefebvre and Smits, 2005; Mackie et al., 2008) hours after initiation of chondrogenic differentiation of ATDC5 cells, Sox9 expression was transiently induced on mRNA (Figure 1A) and protein level (Figure 1B). Sox9 expression increased a second time around day 7 in differentiation (Figure 1A), in parallel with the expression of the important Sox9 transcriptional target Col2a1 (Lefebvre et al., 1997; Figure 1C) and gain of sulfated glycosaminoglycan (GAG) content (Lefebvre and Smits, 2005; Han and Lefebvre, 2008; Figure 1D). The immediate early transient Sox9 expression peak at 2 h in differentiation did not correlate with the induction of expression of well-known Sox9 transcriptional targets such as Col2a1. Hence, we questioned what the function of the early Sox9 expression peak (2 h) was and how it differs from the later Sox9 activity (day 7). We approached this by performing a loss-of-function experiment and comparing the Sox9-dependent transcriptome and proteome in an unbiased manner.

### Transcriptome and Proteome Analysis at 2 Hours and 7 Days in ATDC5 Chondrogenic Differentiation Under Sox9 Knockdown

**FIGURE 2:**
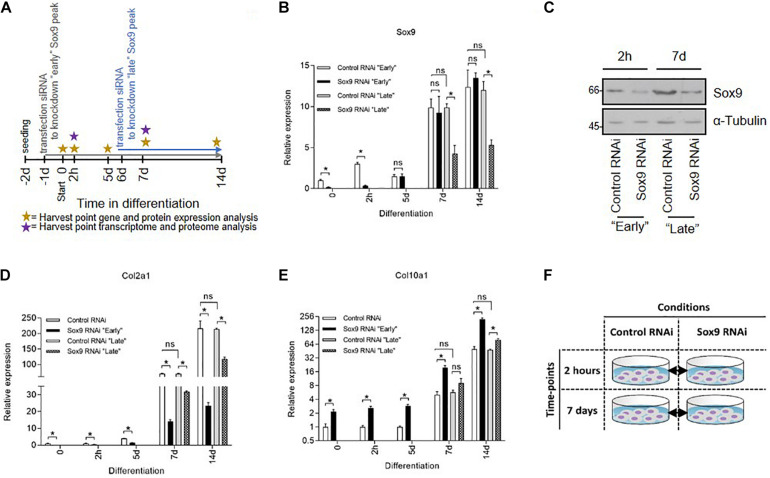
Elucidating the function of early Sox9 expression by transcriptome and proteome analyses. **(A)** Schematic representation of experimental setup for Sox9 knockdown experiment. Specific Sox9 RNAi (100 nM) or scrambled control RNAi (100 nM) were transiently transfected “early” at *t* = −1 day or “late” *t* = 6 day. ATDC5 cells were differentiated from day 0 onward and harvested for transcriptome and proteome analysis at *t* = 0, 2 h, 5 day, 7 day, and 14 day. **(B)** Sox9 mRNA expression during ATDC5 differentiation in Control and Sox9 RNAi conditions (h = hours, d = days) as measured by RT-qPCR. Results were normalized to β-Actin RNA expression and presented relative to *t* = 0. Bars represent mean ± SEM. ns = not significant, *p < 0.05, **p < 0.01, ***p ≤ 0.0001. **(C)** Sox9 protein expression at *t* = 2 h (for “early knockdown condition) and *t* = 7 day (for “late” knockdown conditions) time point in Control and Sox9 RNAi conditions as measured by immunoblotting. α–Tubulin was used as loading control. Molecular weight markers (in kDa) are shown on the left. **(D)** Col2a1 mRNA expression in similar samples from **(B)**. **(E)** Col10a1 mRNA expression in similar samples from **(B)**. **(F)** Schematic representation of time points and comparisons for transcriptomics and proteomics analysis.

To target early Sox9 expression, a siRNA for Sox9 or scrambled control siRNA were transfected prior to initiation of differentiation (*t* = −1 day) (Figure 2A). At *t* = 0, chondrogenic differentiation was induced and cells were differentiated for 14 days. We established effective knockdown of Sox9 at *t* = 0 and *t* = 2 h in differentiation, while at days 5, 7, and 14 in differentiation, Sox9 mRNA levels returned to scrambled siRNA control conditions (Figure 2B; black bars and Figure 2C). In parallel, ATDC5 cells were differentiated and the siRNA for Sox9 or scrambled siRNA was transfected at day 6 in differentiation, to specifically target expression of “late” Sox9 induction. Effective knockdown of Sox9 at day 7 and day 14 was observed at mRNA as well as (Figure 2B; gray bars) at the protein level (Figure 2C). Knockdown of the early Sox9 expression peak resulted in decreased Col2a1 expression, and increased Col10a1 expression at early time points. At days 7 and 14 in ATDC5 differentiation, Sox9 siRNA treatment was not effective anymore (Figure 2B; white versus black bars); however, a major induction of Col2a1 mRNA expression at days 7 and 14 in differentiation was prevented in this condition (Figure 2D). A similar but opposite effect was seen for Col10a1 expression in the early Sox9 peak knockdown condition (Figure 2E). Knockdown of the “late” Sox9 peak (gray bars) also resulted in a decreased Col2a1 and increased Col10a1 expression at days 7 and 14 in differentiation, with a smaller magnitude. These data indicate that the early Sox9 expression peak is paramount for successful chondrogenic differentiation of ATDC5 cells (see Supplementary Figure 1 for confirmation with independent Sox9 siRNA). Differential expression of mRNAs and proteins was determined between the scrambled versus Sox9 siRNA at 2 h as well as at 7 days in ATDC5 differentiation, to target the early and late Sox9 expression peaks (Figure 2F).

**FIGURE 3:**
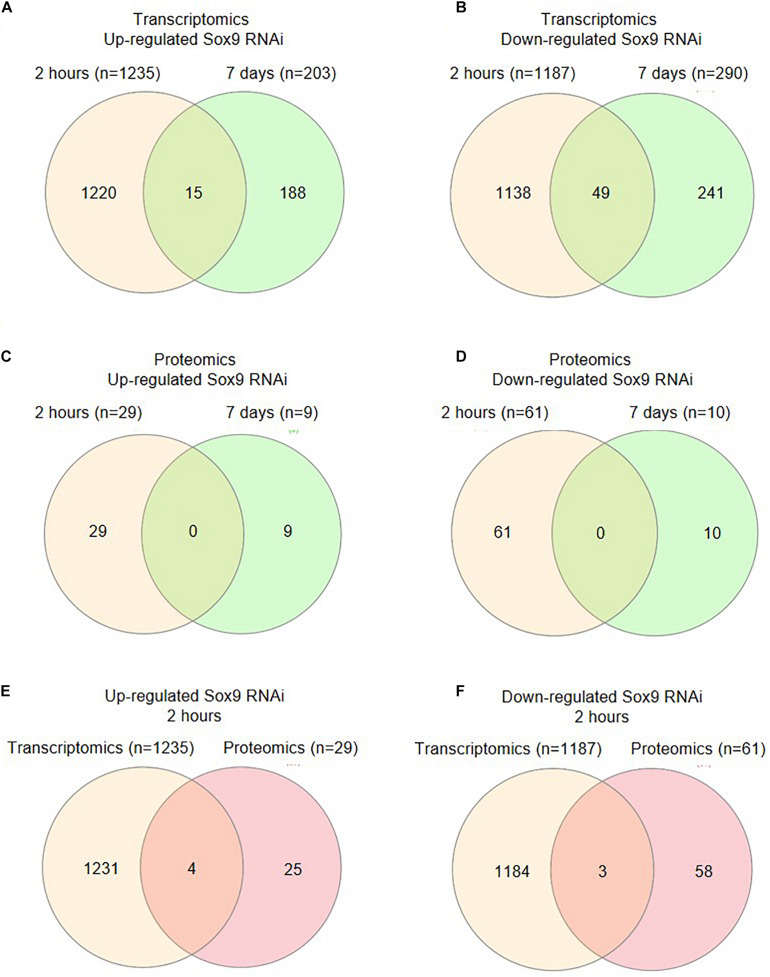
Changes in transcriptome and proteome after Sox9 knockdown. **(A)** Venn diagram (Heberle et al., 2015) showing the numbers of genes whose expression was significantly upregulated upon Sox9 knockdown at 2-h versus 7-day condition. **(B)** Venn diagram showing the numbers of genes whose expression was significantly downregulated upon Sox9 knockdown at 2-h versus 7-day condition. **(C)** Venn diagram showing the numbers of proteins whose expression was significantly upregulated upon Sox9 knockdown at 2-h versus 7-day condition. **(D)** Venn diagram showing the numbers of proteins whose expression was significantly downregulated upon Sox9 knockdown at 2-h versus 7-day condition. **(E)** Venn diagram showing the upregulated genes versus proteins at 2 h in differentiation. **(F)** Venn diagram showing the downregulated genes versus proteins at 2 h in differentiation.

The extracted RNA was used for RNA sequencing and PCA of the transcriptome confirmed that samples from the four groups separated (Supplementary Figure 2). Noteworthy, the separation between the scrambled siRNA and Sox9 siRNA conditions was evident at 2 h, while separation between the scrambled siRNA and Sox9 siRNA conditions at 7 days was less clear. At 2 h in ATDC5 chondrogenic differentiation, knockdown of Sox9 led to the differential expression of 2,422 genes, with 1,235 upregulated genes and 1,187 downregulated genes (Figures 3A,B). At 7 days in differentiation, 493 genes were differentially expressed (203 up and 290 down) due to knockdown of Sox9. From these differentially expressed genes, 203 genes were upregulated (only 15 overlapped with the 2-h time point) and 290 genes were downregulated (49 genes overlapped with the 2-h condition) (Figures 3A,B). All genes that were differentially expressed (FC ≥ 2; *p* < 0.05) at 2 h and at 7 days in ATDC5 chondrogenic differentiation following Sox9 knockdown are shown in Supplementary Tables 2, 3, respectively.

Proteins from control conditions and Sox9 knockdown conditions at 2 h and 7 days in ATDC5 differentiation were quantified using a label-free proteomics approach. PCA plotting confirmed that control samples at 2 h clearly separated from the Sox9 knockdown samples at 2 h in ATDC5 differentiation. Separation between control and Sox9 knockdown conditions was also confirmed at 7 days in chondrogenic differentiation. However, and in concert with the PCA plot of the RNA sequencing data (Supplementary Figure 2), the separation between control and Sox9 knockdown conditions appeared to be most obvious at 2 h in differentiation (Supplementary Figure 3A). At 2 h in differentiation, knockdown of Sox9 caused the differential expression of 90 proteins (29 up and 61 down) (Figures 3C,D and Supplementary Figure 3B). At 7 days in differentiation, the knockdown of Sox9 induced differential expression of 19 proteins (9 up and 10 down). There was no overlap between the Sox9-dependent differentially expressed proteins at 2 h or at 7 days in differentiation (Figures 3C,D and Supplementary Figure 3B). The proteins that were differentially expressed (FC ≥ 2; *p* < 0.05) at 2 h and at 7 days in ATDC5 chondrogenic differentiation following Sox9 knockdown are shown in Supplementary Tables 4, 5, respectively.

These data indicate that at 2 h in chondrogenic differentiation, the knockdown of Sox9 induced different changes in the ATDC5 transcriptome and proteome when compared to knockdown of Sox9 at 7 days in differentiation. In addition, the consequences of Sox9 siRNA treatment appears to be stronger at 2 h than at 7 days of differentiation, as indicated by larger separation in the PCA plots and larger number of differentially expressed genes and proteins. The role of the immediate early Sox9 expression was further investigated by comparing the Sox9-dependent differentially expressed mRNAs and proteins at 2 h in differentiation. Four overlapping mRNAs and proteins are upregulated in the early Sox9 knockdown condition (Rps30/Fau, Avan, Eefsec, and Rpl38; Figures 3E,F and Supplementary Table 6) and three overlapping mRNAs and proteins are downregulated in the early Sox9 knockdown condition (Ube2d3, Dclk1, and Svil; Supplementary Table 6). Except for Rpl38, these overlapping targets are unique for the 2 h in differentiation time point. The relative low number of overlapping genes and proteins (Figures 3E,F) might be explained by the different control of expression regulation at different levels for RNA and protein (Vogel and Marcotte, 2012) and the notion that not all mRNAs are instantaneously translated into protein. Overlapping targets might be involved in a biological process that reacts fastest in a way that is visible in both RNA and protein expression.

### Immediate Early Sox9 Expression Is Involved in Ribosomal Protein Expression

**FIGURE 4:**
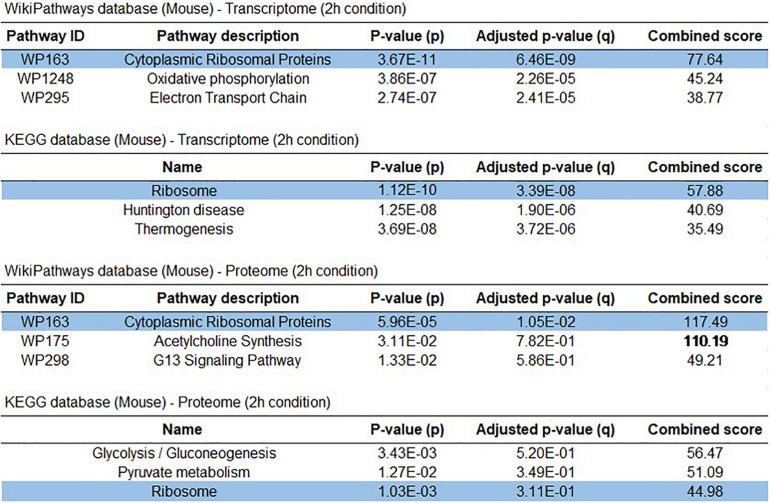
Early Sox9 expression is involved in ribosomal pathways. Top 3 identified enriched pathways from WikiPathway 2019 and KEGG2019 pathway analysis in control RNAi compared to Sox9 RNAi at 2-h condition in ATDC5 differentiation for transcriptome and proteome data sets.

**FIGURE 5:**
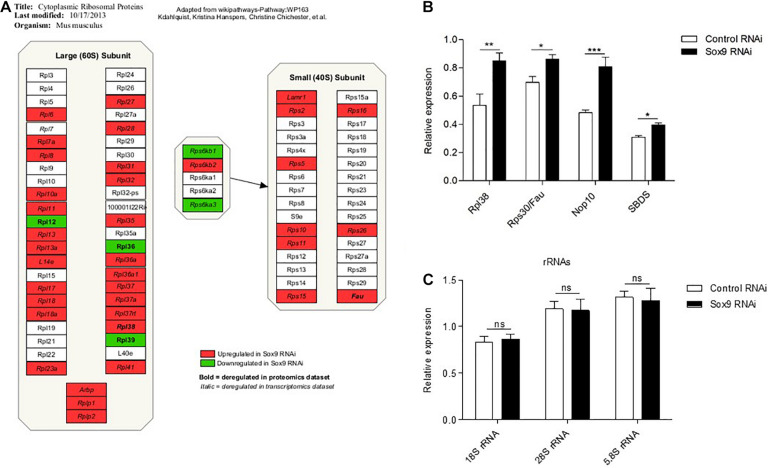
Early Sox9 regulates ribosomal protein expression. **(A)** Schematic representation of significantly differentially expressed ribosomal proteins from transcriptome and proteome datasets based on Wikipathways (Slenter et al., 2018): WP163 using PathVisio 3 (Kutmon et al., 2015). **(B)** Rpl38, Rps30/Fau, Nop10, and SBDS expression at 2 h in ATDC5 differentiation in Control and Sox9 RNAi conditions as measured by RT-qPCR. Results were normalized to β-Actin RNA expression and presented relative to *t* = 0. **(C)** In similar samples from B; 18S rRNA, 28S rRNA, and 5.8S rRNA expression. Bars represent mean ± SEM. ns = not significant, *p < 0.05, **p < 0.01, ***p < 0.0001.

To determine which prominent pathways link to the Sox9-dependent differential transcriptome and proteome at 2 h in differentiation, we performed pathway analyses. Two independent Pathway analyses revealed that “Cytoplasmic Ribosomal Proteins” and “Ribosome” pathways were in the top three of identified enriched pathways (Figure 4). This strong overrepresentation of the “Cytoplasmic Ribosomal Proteins” and “Ribosome” pathways was not obvious in the Sox9-dependent differential transcriptome and proteome at day 7 in differentiation (Supplementary Table 7). Further analysis revealed the differential expression of 29 ribosomal protein encoding genes from the large (60S) ribosomal subunit (Rpls) and 10 ribosomal proteins from the small (40S) ribosomal subunit (Rpss) in the Sox9 knockdown condition at 2 h in ATDC5 chondrogenic differentiation (Figure 5A and Supplementary Table 2). In addition, the 2-h proteomics datasets demonstrated the differential expression of five ribosomal Rpl and Rps proteins (Figure 5A and Supplementary Table 4). Notably, the four overlapping mRNAs and proteins (Rps30/Fau, Avan, Eefsec, and Rpl38) that were upregulated in the Sox9 knockdown condition at 2 h in ATDC5 chondrogenic differentiation (Figures 3E, 5B, Supplementary Figure 4A) are all linked to protein translation and represent either ribosomal protein subunits or factors with a known function in ribosome biogenesis. Additional factors involved in ribosome biogenesis, but only differentially expressed in either the 2-h transcriptomics or proteomics datasets were SBDS, Nop10, and Brix1 (Figure 5B and Supplementary Tables 2, 3). Where expression of Sox9 was significantly increased at 2 h in ATDC5 differentiation, expression of Rpl38, Rps30/Fau, Nop10, and SBDS was significantly decreased compared to t0 (Supplementary Figure 4C). Since ribosomes do not only consist of proteins, but depend on structural and catalytically active ribosomal RNAs (rRNAs), we investigated whether rRNA levels were affected by the Sox9 knockdown at 2 h in differentiation in ATDC5. Expression of 18S rRNA, 28S rRNA, and 5.8S rRNA was not significantly different between the conditions (Figure 5C) (see Supplementary Figure 4B for confirmation with independent Sox9 siRNA). Together, these data indicate that the Sox9 expression during early ATDC5 chondrogenic differentiation is involved in expression of ribosomal proteins and proteins involved in ribosome biogenesis.

### Early Sox9 Expression Regulates Protein Translation Capacity and Ribosome Translation Modus

**FIGURE 6:**
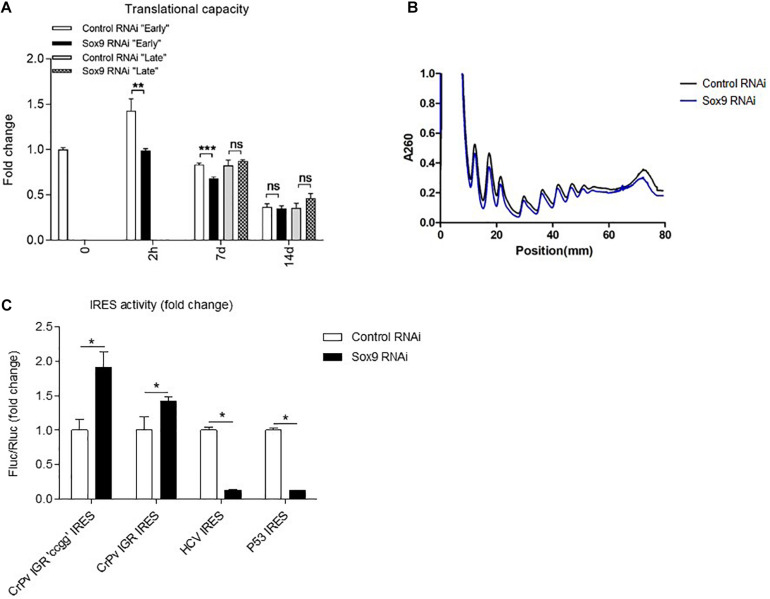
Knockdown of early Sox9 expression leads to a reduced translational capacity and lower amount of ribosomes and alters ribosome modus. **(A)** Total protein translation measurements based on puromycin incorporation was normalized to total DNA content per well (mean ± SEM, *n* = 6/group) at indicated time points. **(B)** Polysome fractionation of control and Sox9 knockdown cells (mean only, *n* = 3/group) after 2 h of differentiation. **(C)** Ribosome modus was assessed for the CrPv IGR CCGG mutant IRES, the intact CrPv IGR IRES, the HCV IRES, and the P53 IRES after 24 h of differentiation. Bars represent mean ± SEM. ns = not significant, *p < 0.05, **p < 0.01, ***p ≤ 0.0001.

**FIGURE 7:**
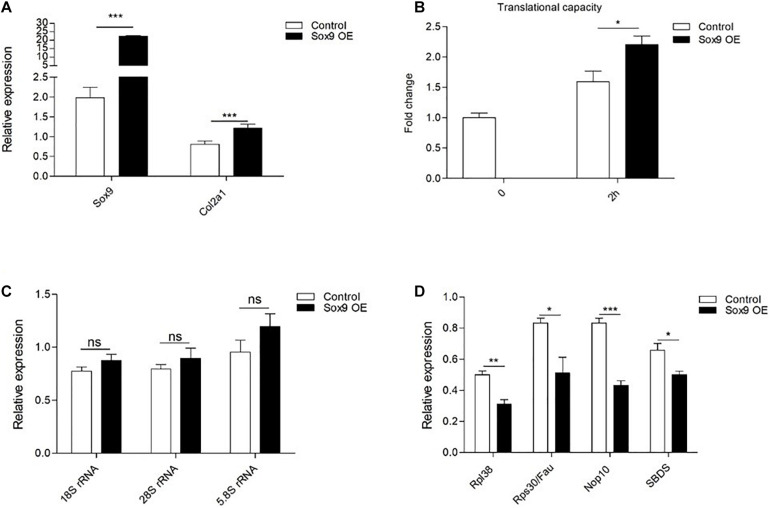
Overexpression of early Sox9 leads to an increased translational capacity. **(A)** Sox9 mRNA expression at 2 h in ATDC5 differentiation in Control and Sox9 overexpression (OE) conditions as measured by RT-qPCR. Results were normalized to β-Actin RNA expression and presented relative to *t* = 0. **(B)** Total protein translation measurements based on puromycin incorporation was normalized to total DNA content per well (mean ± SEM, *n* = 6/group) at indicated time points. **(C)** In similar samples from A; 18S rRNA, 28S rRNA, and 5.8S rRNA expression at 2 h in ATDC5 differentiation in Control and Sox9 overexpression conditions. **(D)** In similar samples from A; *Rpl38, Rps30/Fau, Nop10*, and SBDS expression at 2 h in ATDC5 differentiation in Control and Sox9 overexpression conditions. Bars represent mean ± SEM. ns = not significant, *p < 0.05, **p < 0.01, ***p ≤ 0.0001.

Since we identified the differential expression of ribosomal protein subunits and ribosome biogenesis factors, combined with unaltered rRNA expression levels, we hypothesized that ribosomes of early Sox9 knockdown ATDC5 cells are functionally distinct. To address this, we measured total translational capacity, performed polysome fractionation, and evaluated ribosome translation modus in Sox9 knockdown and control ATDC5 cells. Following the knockdown of immediate early Sox9 expression, a reduction of the total protein translational capacity was observed at 2 h in chondrogenic differentiation (Figure 6A). The abrogation of early Sox9 expression also caused a reduction of total protein translational capacity at day 7 in differentiation [while Sox9 levels normalized at 7 days following the early knockdown (Figure 2B)]. This impact on translation capacity was lost at day 14 in differentiation (Figure 6A). In contrast, late knockdown of Sox9 expression did not affect ATDC5 translational capacity at 7 days in chondrogenic differentiation. This is consistent with transcriptome and proteome data. To assess if Sox9 knockdown had a specific effect on polysomal distribution of ribosomes, we performed sucrose density gradient separation of ribosomal subunits. Knockdown of the immediate early Sox9 expression resulted in an overall lower abundance of ribosomal subunits, which is in agreement with reduced translational activity (Figure 6B). In addition to total ribosome translation capacity (Figure 6A), the modus of translation is also subject to regulation. Thus, we evaluated the activity of IRES (internal ribosome entry site)- over cap-mediated protein translation using well-characterized bicistronic reporter constructs (CrPv IGR, HCV, and P53 IRES) (Collier et al., 1998; Ray et al., 2006; Deniz et al., 2009). We observed a 1.5-fold induction of the ITAF (IRES *trans-*acting factor) independent CrPv IGR IRES activity and 5-fold down regulation of both the HCV and P53 IRES activity in Sox9 knockdown ATDC5 cells compared to controls (Figure 6C, and Supplementary Figure 5). We next investigated whether overexpression of Sox9 levels during early ATDC5 differentiation may have a reciprocal effect on the translation capacity as opposed to the Sox9 knockdown conditions. Overexpression of Sox9 was confirmed (Figure 7A), and the mRNA expression of the Sox9 transcriptional target Col2a1 was significantly induced by Sox9 overexpression (Figure 7A). The overexpression of Sox9 resulted in a significant increase in translational capacity at 2 h in ATDC5 differentiation (Figure 7B). This increase in translational capacity in the Sox9 overexpression condition was not accompanied by an increase in expression of rRNAs (Figure 7C). Contrary to the knockdown of Sox9 (Figure 5B), expression of ribosomal protein subunits Rpl38 and Rps30/Fau was significantly downregulated when Sox9 was overexpressed (Figure 7D). This was also the case for ribosome biogenesis factors Nop10 and SBDS (Figure 7D).

Taken together, knockdown of early Sox9 expression during ATDC5 chondrogenic differentiation reduced protein translational activity over a 7-day time period, which was reflected by a general reduction of A_260_ signal in a polysome fractionation experiment at 2 h in differentiation. This was associated with a differential effect on ribosome translation modus during the first day of differentiation. Sox9 overexpression during early ATDC5 differentiation was able to induce reciprocal effects of the Sox9 knockdown on total protein translation.

## Discussion

Early chondrogenic lineage commitment during mesenchymal condensation is driven by Sox9 (Akiyama et al., 2002; Liu et al., 2018; Lefebvre et al., 2019). Following lineage commitment, Sox9 is a key regulator of cartilage ECM synthesis, by driving expression of key ECM molecules such as Acan, Col2a1, and others (Lefebvre et al., 1997, 1998, 2001; Akiyama et al., 2002; Jenkins et al., 2005; Rentsendorj et al., 2005; Genzer and Bridgewater, 2007; Han and Lefebvre, 2008; Oh et al., 2010). Sox9 also safeguards maintenance of articular cartilage homeostasis by influencing chondrocyte Nkx3-2 levels, a transcriptional repressor of chondrocyte hypertrophy (Yamashita et al., 2009; Caron et al., 2015). This dual action of Sox9 is recapitulated in *in vitro* models of chondrogenic differentiation, as we have previously reported on bi-phasic expression dynamics of Sox9 in ATDC5 and bone marrow-derived stem cells (BMSC) chondrogenic differentiation (Caron et al., 2012; Spaapen et al., 2013). In this earlier work, we demonstrated that early (hours) transient induction of Sox9 expression driven by NFκB/p65 or Egr1 in ATDC5 chondrogenic differentiation is a prerequisite for late-stage (days) expression of cartilage ECM genes. As a key transcription factor for cartilage, the vast majority of investigations on the downstream functions of Sox9 have mainly focused on its role in the transcriptional regulation of cartilage ECM genes (Lefebvre et al., 1997, 1998, 2001; Akiyama et al., 2002; Jenkins et al., 2005; Rentsendorj et al., 2005; Genzer and Bridgewater, 2007; Han and Lefebvre, 2008; Oh et al., 2010). In addition, a function in epigenetic reprogramming has been suggested for early Sox9 (Spaapen et al., 2013), as well as a role for Sox9 in activating super-enhancers in chondrocytic cells (Liu and Lefebvre, 2015). However, its downstream cell biological consequences during early chondrogenic differentiation are incompletely understood. The present study demonstrates that Sox9 expression during the very early phase of ATDC5 chondrogenic differentiation regulates the expression of ribosomal protein subunits, as well as proteins that are involved in ribosome biogenesis that together modulate ribosome activity and translation modus. These data, for the first time, connect the Sox9 transcription factor to regulation of protein translation during chondrogenic differentiation.

Ribosomopathies are severe genetic diseases caused by mutations in genes involved in ribosome biogenesis and function and are, among others, associated with developmentally related skeletal malformations, caused by impairment of chondrogenic development of the growth plates (Trainor and Merrill, 2014; Venturi and Montanaro, 2020). This indicates that chondrogenic differentiation is particularly susceptible to disturbances in ribosome protein translation activity. Indeed, during chondrogenesis, a large amount of cartilage ECM is produced by the developing growth plate and disturbances in ribosome activity are likely to impair ECM synthesis, with consequences for the development of skeletal elements. It should, however, be noted that the link between Sox9 and chondrocyte translation activity in the current work was particularly present during early rather than late differentiation. This is highlighted by the deregulated expression of ribosomal subunits following Sox9 knockdown in early ATDC5 chondrogenic differentiation (Figures 4, 5). In addition, the knockdown of Sox9 specifically impacted total protein translation throughout differentiation upon early knockdown, while not having an effect on protein translation when knocked down during later in chondrogenic differentiation (Figure 6). The link between early Sox9 and chondrocyte protein translation suggests that protein translation is likely to be paced through Sox9 during early chondrogenic differentiation. However, it remains to be determined how early Sox9 is specifically able to influence chondrocyte translational capacity. Our present data suggest that expression of ribosomal protein subunits, ribosome biogenesis factors, and ancillary ribosomal factors (such as ITAFs) depends on Sox9 during early chondrogenic differentiation, with downstream consequences for translation in the later differentiation program. Since we found these ribosomal genes and proteins differentially expressed after Sox9 knockdown, we studied the supplementary data of previously published Sox9 ChIP-seq and Sox9^–/–^ mouse studies (Liu and Lefebvre, 2015; Liu et al., 2018). In supplementary data of a Sox9 ChIP-seq study, we found 24 Rps and Rpl genes that were enriched in Sox9 occupancy and 14 with Sox6 occupancy of which 10 overlapped (Liu and Lefebvre, 2015). Notably, this included Rpl38, which we found to be differentially expressed at the mRNA and protein level in our present Sox9 knockdown condition.

Aside from ribosome core components, we identified Sox9-dependent differential expression of several factors regulating the mode of protein translation. The rate-limiting step of cap-mediated translation is eukaryotic initiation factor 4 (Svitkin et al., 2005). Interestingly, EIF4BP2 was downregulated at the protein level at 2 h of differentiation in Sox9 knockdown cells. EIF4 binding proteins were shown to regulate cell proliferation, but not cell size (Dowling et al., 2010). Unexpectedly, we found strong differences in IRES activity upon early Sox9 knockdown after 24 h of differentiation. Of note, the CrPv IGR IRES [a type IV IRES (Johnson et al., 2017)] does not require ITAFs and is able to recruit the ribosome directly for translation (Jan and Sarnow, 2002). The activity of this IRES was increased and might be regulated by specific Rps/Rpls that transiently interact with the core ribosome components. The HCV (a type III IRES) and P53 IRES do require additional ITAF co-factors (Godet et al., 2019). Based on the increased expression of the ITAF Rpl38 (Xue et al., 2015), it is tempting to speculate that other ITAFs that facilitate HCV/P53 IRES translation are downregulated and may contribute to alternative use of ribosome translation modus. Rpl10a, Rpl11, Rpl38, and Pdcd4 were shown to regulate IRES-mediated translation (Godet et al., 2019). Rpl10a is known to activate the *IGF2*, *APP*, *Chmp2A*, and *Bcl-2* IRESs. Gain- and loss-of-function studies in *Drosophila* showed that *Rpl10a* regulates insulin signaling (Chaichanit et al., 2018). Moreover, *Rpl10a* was found to be preferentially translated by a sub pool of ribosomes in embryonic stem cells that required ribosome-associated Rpl10a (Shi et al., 2017). Insulin and insulin-like growth factor I (IGF1) signaling are crucial for ATDC5 differentiation (Atsumi et al., 1990). Rpl11 induced the *BAG1*, *CSDE1*, and *LamB1* IRESs, while *Rpl38* activated a Hox gene IRES (Horos et al., 2012; Xue et al., 2015). Upregulation of *Rpl11* led to stabilization of P53 and reduced proliferation of breast cancer cell lines (Tong et al., 2020). In contrast, we found a reduction in *P53* IRES activity. Of note, P53 and ribosome biogenesis were recently coupled through SBDS (Ribosome Maturation Factor) (Tong et al., 2020). In our dataset, SBDS was downregulated at the protein level at 2 h in differentiation in Sox9 siRNA-treated cells, which matches the observed reduction in ribosomes and/or ribosome activity. Pdcd4 activated or inhibited the P53, INR, IGF1R, BcL-XL, and XIAP IRESs (Godet et al., 2019). The IGF1R IRES might again be relevant for the ATDC5 differentiation model. Finally, Rpl38 was the only ITAF that was found to be upregulated at both the mRNA and protein level. It was found to control HOX gene translation during murine embryonic development (Xue et al., 2015) and knockout led to ectopic mineralization in certain soft tissues (Noben-Trauth and Latoche, 2011).

We identified multiple connections between immediate early Sox9 expression during ATDC5 chondrogenic differentiation and downstream consequences for expression of ribosomal protein subunits, ribosome biogenesis factors, and ITAFs. The connection between Sox9 expression and protein translation appears to be centered in early chondrogenic differentiation, with consequences for protein translation in later stage of chondrogenic differentiation. This suggests a role for early Sox9 in the priming of progenitor cells in the chondrogenic differentiation program for cartilaginous ECM production later in differentiation. This provides a new level of understanding how Sox9 controls the fate of chondrogenic differentiation at the level of protein synthesis. In this respect, it is tempting to speculate on the classification of campomelic dysplasia (OMIM #114290) (Mortier et al., 2019), as links between Sox9 and genes involved in ribosomopathies (Nakhoul et al., 2014; Trainor and Merrill, 2014) were identified in the present work (SBDS, Rpl11, and Rps26). In conclusion, we collected essential new data on the regulation by Sox9 during early chondrogenic differentiation, uncovering an unanticipated role of Sox9 in ribosome biogenesis and protein translational capacity.

## Data Availability Statement

RNA-seq data have been deposited in the ArrayExpress database at EMBL-EBI (www.ebi.ac.uk/arrayexpress) under accession number E-MTAB-10333. Proteomics data can be found in the ProteomeXchange Consortium via the PRIDE (37) partner repository with the dataset identifier PXD024715. All other data used and/or analyzed during the current study are available from the corresponding author on reasonable request.

## Author Contributions

MC, ME, BC-P, RH, LR, GA, and TW: substantial contributions to research design. MC, ME, BC-P, BH, KD, AC, and GA: substantial contributions to the acquisition of samples. MC, ME, BC-P, BH, KD, AC, MP, GA, and TW: substantial contributions to analysis. MC, ME, BC-P, RH, BH, KD, AC, MP, LR, GA, and TW: substantial contributions to the interpretation of data, revising manuscript critically, approval of the submitted and final versions. MC, ME, BC-P, GA, and TW: drafting the manuscript. All authors contributed to the article and approved the submitted version.

## Conflict of Interest

MC and TW are inventor on patents WO2017178251 and WO2017178253 (Licensed to Chondropeptix). LR and TW have shares in Chondropeptix and are CDO and CSO of Chondropeptix. The remaining authors declare that the research was conducted in the absence of any commercial or financial relationships that could be construed as a potential conflict of interest.

## Footnotes

1 www.ebi.ac.uk/arrayexpress
