# Supplementary material for: Sox9 Determines Translational Capacity During Early Chondrogenic Differentiation of ATDC5 Cells by Regulating Expression of Ribosome Biogenesis Factors and Ribosomal Proteins
Source: Front Cell Dev Biol. 2021 Jun 21;9:686096. doi: 10.3389/fcell.2021.686096 (PMC8256280; doi:10.3389/fcell.2021.686096)
Supplement: Supplementary file 1 [file Data_Sheet_1.docx]

**Sox9 determines translational capacity during early chondrogenic differentiation of ATDC5 cells by regulating expression of ribosome biogenesis factors and ribosomal proteins**

**Marjolein M.J. Caron^1†*^, Maxime Eveque^2†^, Berta Cillero-Pastor^2^, Ron M. A. Heeren^2^, Bas Housmans^1^, Kasper Derks^3^, Andy Cremers^1^, Mandy J. Peffers^4^, Lodewijk W. van Rhijn^1^, Guus van den Akker^1†^, Tim J.M. Welting^1†^**

^1^Laboratory for Experimental Orthopedics, Department of Orthopedic Surgery, CAPHRI Care and Public Health Research Institute, Maastricht University Medical Center. P.O. Box 5800, 6202 AZ, Maastricht, the Netherlands.

^2^Maastricht MultiModal Molecular Imaging institute (M4I), Division of Imaging Mass Spectrometry, Maastricht University Medical Center. P.O. Box 5800, 6202 AZ, Maastricht, the Netherlands.

^3^Department of Clinical Genetics, Maastricht University Medical Center. P.O. Box 5800, 6202 AZ, Maastricht, the Netherlands.

^4^Department of Musculoskeletal Biology, Institute of Life Course and Medical Sciences, University of Liverpool, Liverpool, United Kingdom

^†^ contributed equally

**Supplemental Files**

**Supplementary Table 1: Primer sequences for RT-qPCR**
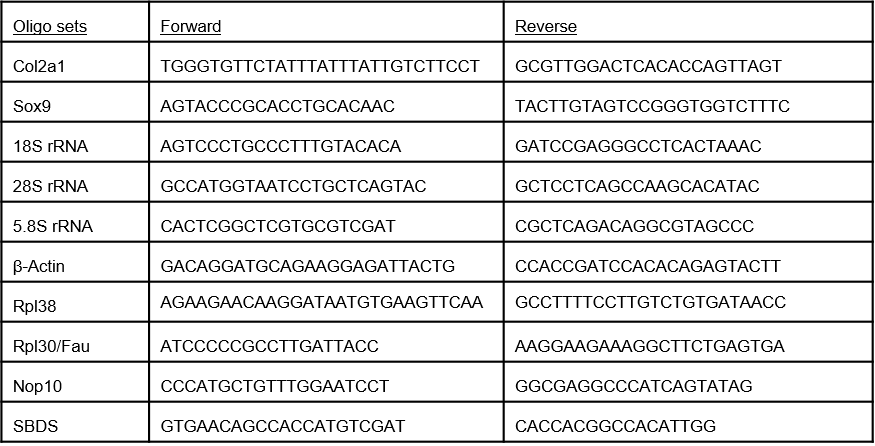


The 5’ to 3’ forward and reverse oligonucleotide sequences (Mus musculus) used for RT-qPCR are listed in the table.

**Supplementary Table 2: RNA sequencing at t=2h in ATDC5 differentiation**

- Excelfile

**Supplementary Table 3: RNA sequencing at t=7d in ATDC5 differentiation**

- Excelfile

**Supplementary Table 4: Nano LC-ESI-MS/MS proteomics differential expressed targets at t=2h in ATDC5 differentiation**

- Excelfile

**Supplementary Table 5: Nano LC-ESI-MS/MS proteomics differential expressed targets at t=7d in ATDC5 differentiation**

- Excelfile

**Supplementary Table 6: Up- and downregulated genes versus proteins at 2 hours in differentiation.**

- Excelfile

**Supplementary Table 7: Top 3 identified enriched pathways in control RNAi compared to Sox9 RNAi at 7 days in ATDC5 differentiation**

Top 3 identified enriched pathways from WikiPathway 2019 and KEGG2019 pathway analysis in control RNAi compared to Sox9 RNAi at 7 days condition in ATDC5 differentiation for transcriptome and proteome data sets.


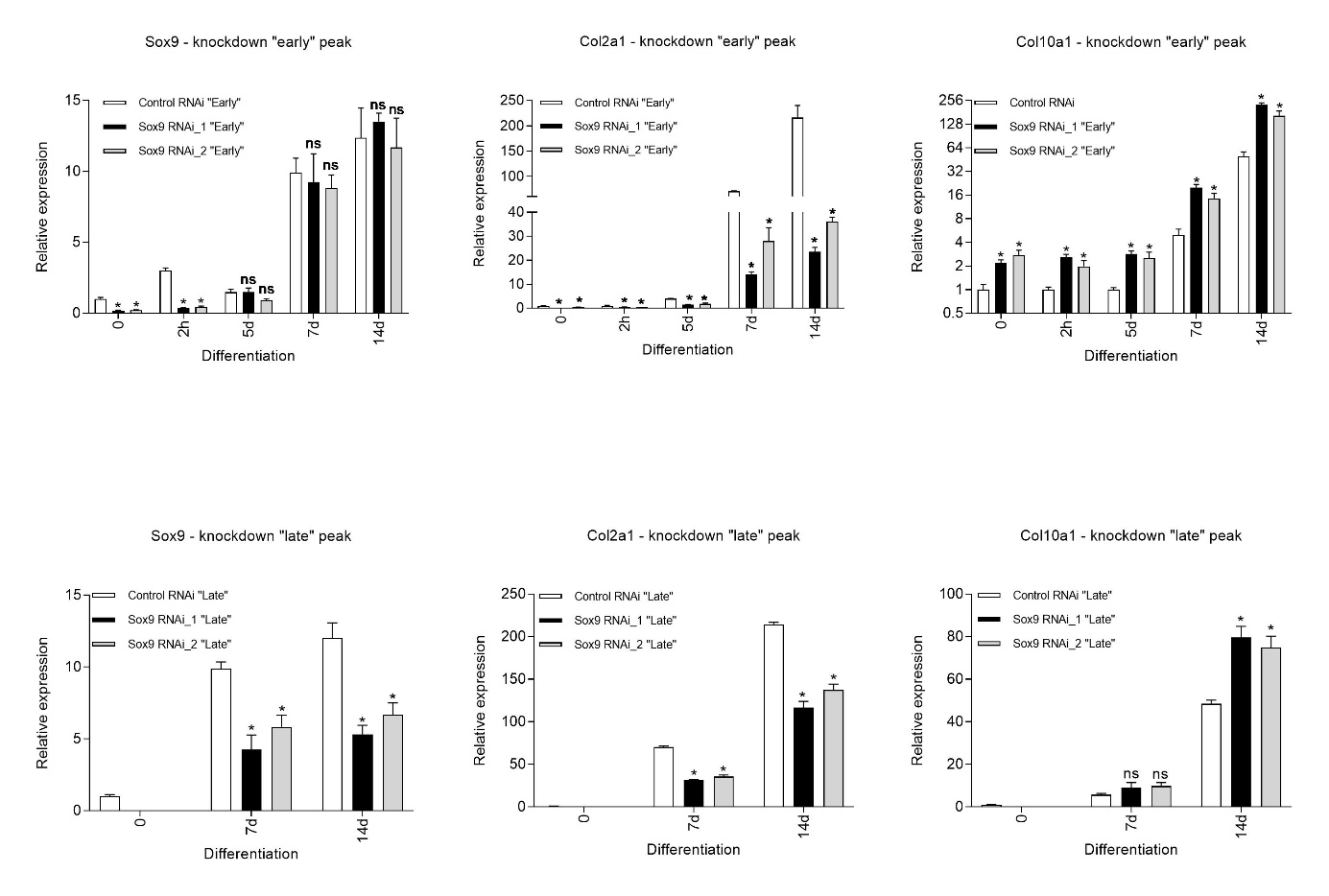


F

E

D

B

C

A

**Supplementary Figure 1: Influence of early and late Sox9 expression peaks at chondrogenic differentiation**

Specific Sox9 RNAi (100 nM; 2 different siRNA’s) or scrambled control RNAi (100 nM) were transiently transfected “early” at t=-1d or “late” t=6d to knockdown the “early” or “late” Sox9 expression peaks (Figure 1). Sox9 RNAi_1: sense: 5’-GACUCACAUCUCUCCUAAUTT-3’, anti-sense: 5’-AUUAGGAGAGAUGUGAGU

CTT-3’. Sox9 RNAi_2: sense: 5’-CUCCACCUUCACUUACAUGTT-3’, anti-sense: 5’-CAUGUAAGUGAAG

GUGGAGTT-3’. ATDC5 cells were differentiated from day 0 onwards and harvested for gene expression analysis at t=0, 2h, 5d, 7d, 14d. **A/D**: Sox9 mRNA expression during ATDC5 differentiation in Control and both Sox9 RNAi conditions (h=hours, d=days) as measured by RT-qPCR. Results were normalized to β-Actin RNA expression and presented relative to t=0. **B/E**: Col2a1 mRNA expression in similar samples from (A). **C/F**: Col10a1 mRNA expression in similar samples from (A). Bars represent mean±SEM. ns= not significant, *=p<0.05, **=p<0.01, p=<0.0001.


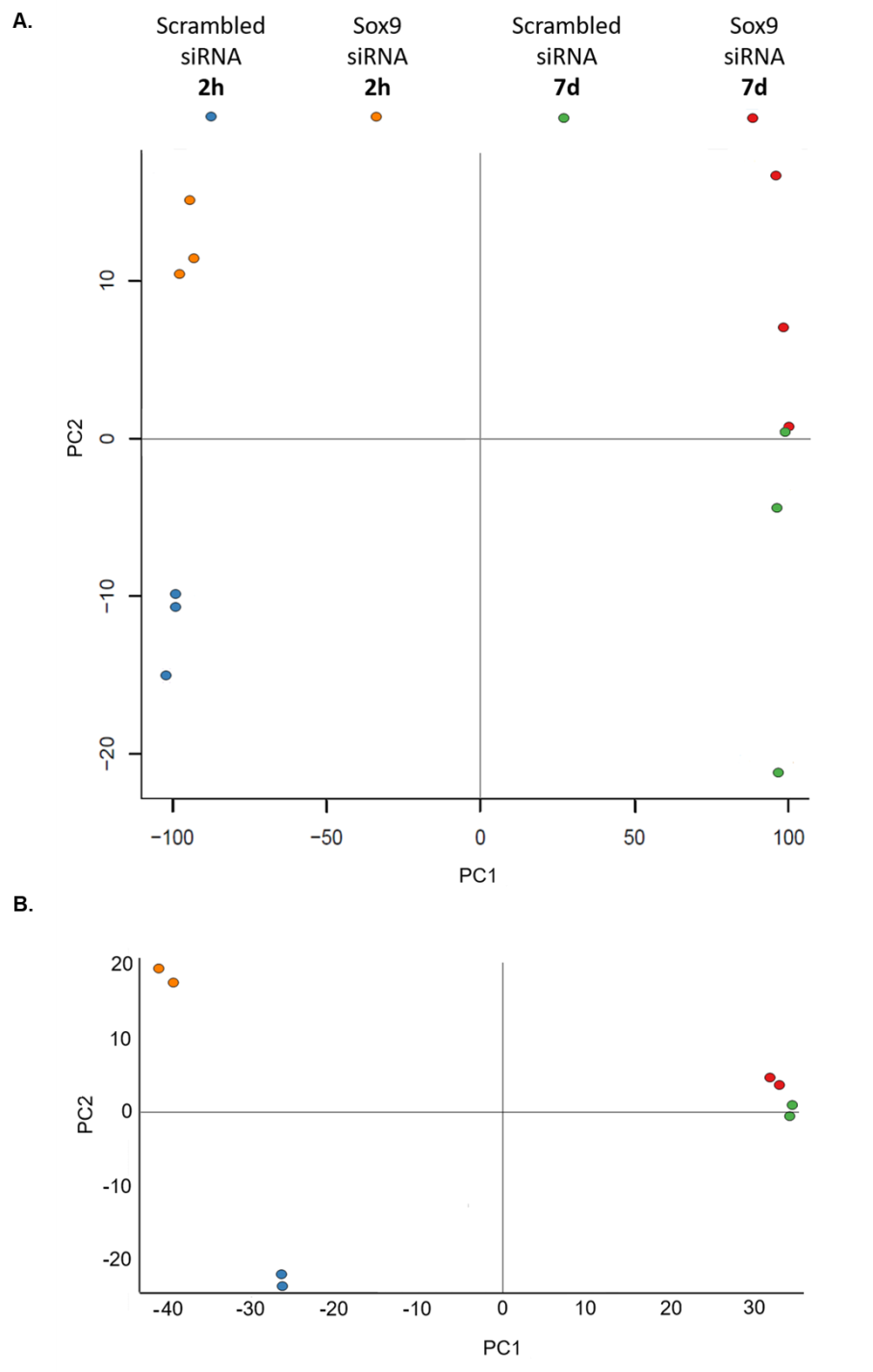


(1,6%)

(92,7%)

**Supplementary Figure 2: PCA plot RNAseq dataset**

PCA plot RNAseq data ATDC5 diff T=2h Control siRNA (100nM) vs T=2h Sox9 siRNA (100nM) and T=7d Control siRNA (100nM) vs T=7d Sox9 siRNA (100nM)


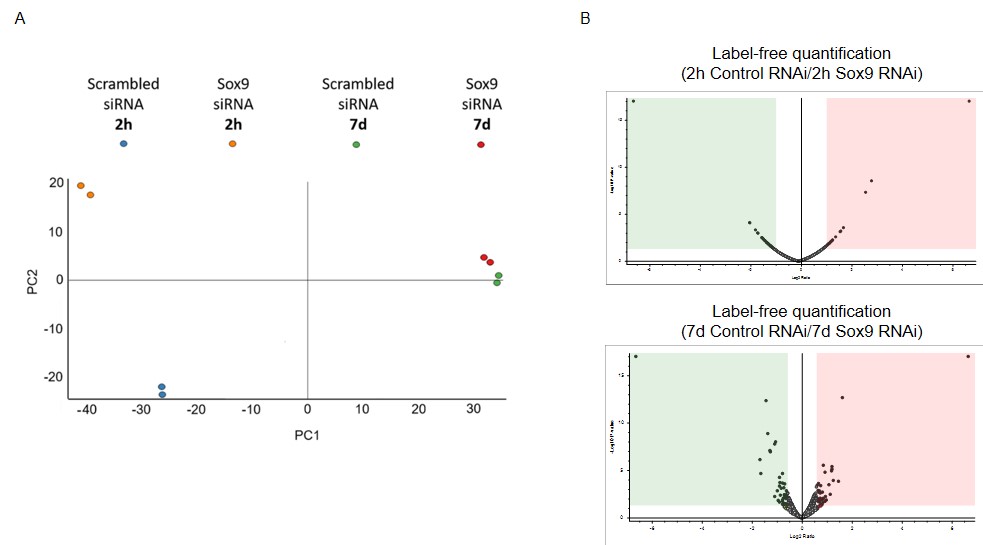


(68,6%)

(13,4%)

**Supplementary Figure 3: Specification of proteomics dataset**

**A**: PCA plot proteomica dataset of ATDC5 differentiation at T=2h Control siRNA (100nM) vs T=2h Sox9 siRNA (100nM) and T=7d Control siRNA (100nM) vs T=7d Sox9 siRNA (100nM) **B:** Label-free quantification of proteomica data of ATDC5 differentiation at T=2h control siRNA (100nM) vs T=2h Sox9 siRNA (100nM) and T=7d Control siRNA (100nM) vs T=7d Sox9 siRNA (100nM)


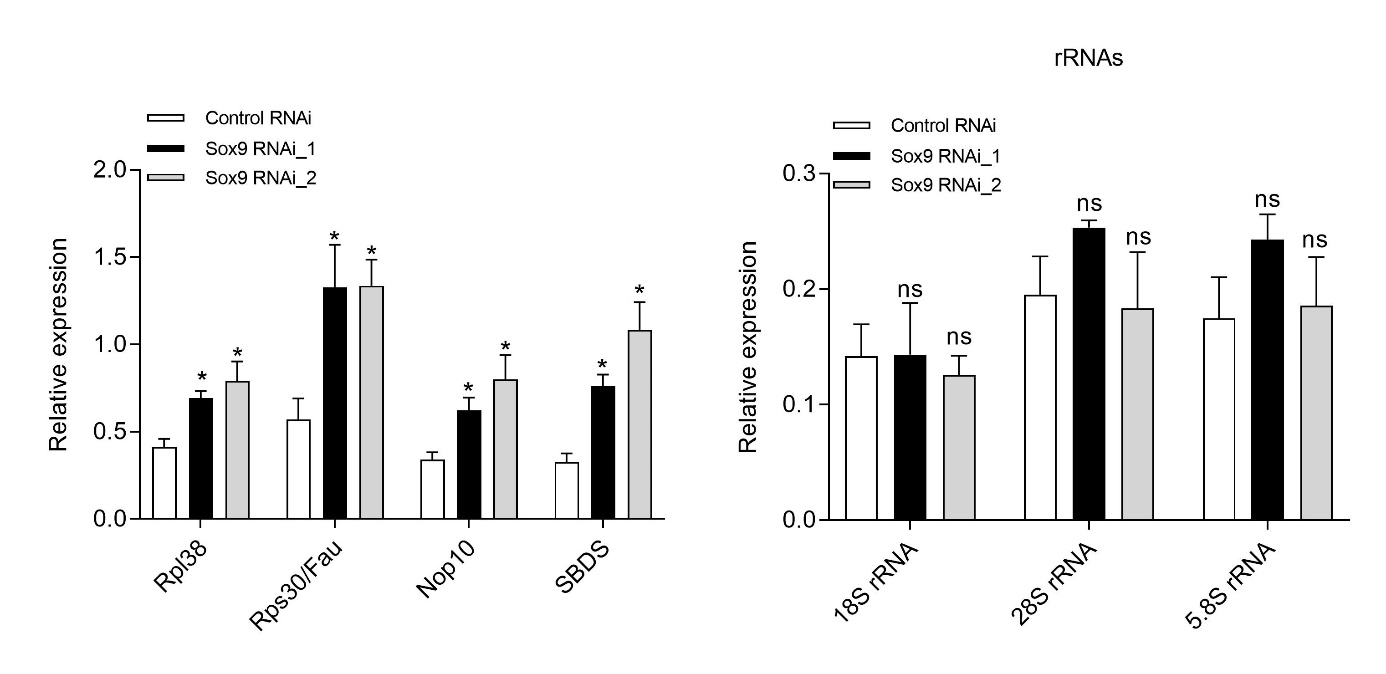


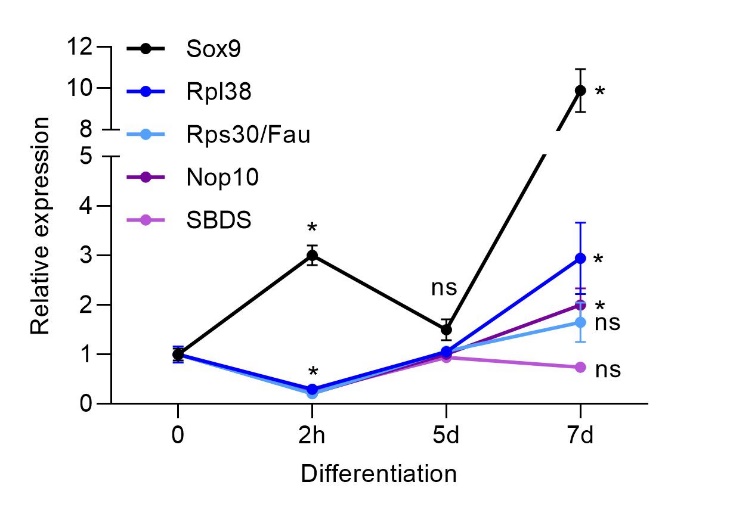


**Supplementary Figure 4: Early Sox9 regulates ribosomal protein expression**

Specific Sox9 RNAi (100 nM; 2 different siRNA’s) or scrambled control RNAi (100 nM) were transiently transfected “early” at t=-1d to knockdown the “early” Sox9 expression peaks (Figure 1). Sox9 RNAi_1: sense: 5’-GACUCACAUCUCUCCUAAUTT-3’, anti-sense: 5’-AUUAGGAGAGAUGUGAGUCTT-3’. Sox9 RNAi_2: sense: 5’-CUCCACCUUCACUUACAUGTT-3’, anti-sense: 5’-CAUGUAAGUGAAGGUGGAGTT-3’. ATDC5 cells were differentiated from day 0 onwards and harvested for gene expression analysis at 2h. **A**: Rpl38, Rps30/Fau, Nop10 and SBDS expression at 2 hours in ATDC5 differentiation in Control and Sox9 RNAi conditions as measured by RT-qPCR. Results were normalized to β-Actin RNA expression and presented relative to t=0. **B**: In similar samples from A; 18S rRNA, 28S rRNA and 5.8S rRNA expression. **C**: ATDC5 cells were differentiated from day 0 onwards and harvested for gene expression analysis at 0, 2 hours, 5 days and 7 days. Gene expression of Sox9, Rpl38, Rps30/Fau, Nop10 and SBDS was determined over differentiation time. Bars represent mean±SEM. ns= not significant, *=p<0.05, **=p<0.01, ***=p<0.0001.


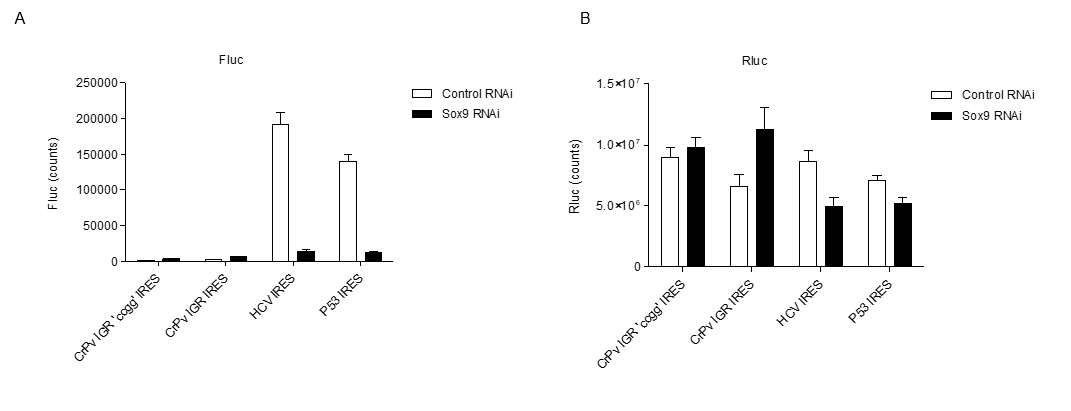


**Supplementary Figure 5: Raw data of individual cistrons in the bicistronic reporter assay.**

Ribosome modus was assessed at 24 hours of differentiation and two days after transfection. **A**: Raw firefly luciferase counts following reporter plasmid transfections. **B**: Raw renilla luciferase counts following reporter plasmid transfections. Mean±SEM, n=3/group.
